# An Analysis of the Global Expression of MicroRNAs in an Experimental Model of Physiological Left Ventricular Hypertrophy

**DOI:** 10.1371/journal.pone.0093271

**Published:** 2014-04-21

**Authors:** Nidiane C. Martinelli, Carolina R. Cohen, Kátia G. Santos, Mauro A. Castro, Andréia Biolo, Luzia Frick, Daiane Silvello, Amanda Lopes, Stéfanie Schneider, Michael E. Andrades, Nadine Clausell, Ursula Matte, Luis E. Rohde

**Affiliations:** 1 Experimental and Molecular Cardiovascular Laboratory and the Heart Failure and Cardiac Transplant Unit from the Cardiology Division at Hospital de Clínicas de Porto Alegre, Porto Alegre, RS, Brazil; 2 Post-Graduate Program in Cardiology and Cardiovascular Science, Porto Alegre, RS, Brazil; 3 Post-Graduate Program in Genetics and Molecular Biology at the Federal University of Rio Grande do Sul, Porto Alegre, RS, Brazil; University of Otago, New Zealand

## Abstract

**Background:**

MicroRNAs (miRs) are a class of small non-coding RNAs that regulate gene expression. Studies of transgenic mouse models have indicated that deregulation of a single miR can induce pathological cardiac hypertrophy and cardiac failure. The roles of miRs in the genesis of physiological left ventricular hypertrophy (LVH), however, are not well understood.

**Objective:**

To evaluate the global miR expression in an experimental model of exercise-induced LVH.

**Methods:**

Male Balb/c mice were divided into sedentary (SED) and exercise (EXE) groups. Voluntary exercise was performed on an odometer-monitored metal wheels for 35 days. Various tests were performed after 7 and 35 days of training, including a transthoracic echocardiography, a maximal exercise test, a miR microarray (miRBase v.16) and qRT-PCR analysis.

**Results:**

The ratio between the left ventricular weight and body weight was increased by 7% in the EXE group at day 7 (p<0.01) and by 11% at day 35 of training (p<0.001). After 7 days of training, the microarray identified 35 miRs that were differentially expressed between the two groups: 20 were up-regulated and 15 were down-regulated in the EXE group compared with the SED group (p = 0.01). At day 35 of training, 25 miRs were differentially expressed: 15 were up-regulated and 10 were decreased in the EXE animals compared with the SED animals (p<0.01). The qRT-PCR analysis demonstrated an increase in miR-150 levels after 35 days and a decrease in miR-26b, miR-27a and miR-143 after 7 days of voluntary exercise.

**Conclusions:**

We have identified new miRs that can modulate physiological cardiac hypertrophy, particularly miR-26b, -150, -27a and -143. Our data also indicate that previously established regulatory gene pathways involved in pathological LVH are not changed in physiological LVH.

## Introduction

Physiological cardiac hypertrophy is a common adaptation that occurs in the heart during exercise training and leads to morphological changes without overall ventricular dysfunction [1], [2]. The cellular and molecular mechanisms involved in the genesis of physiologic cardiac hypertrophy are not as well understood as those implicated in pathological growth, but both processes require the activation of a specific set of genes responsible for cardiomyocyte expansion [3], [4], [5]. Studies that unravel the molecular mechanisms underlying the changes that occur during physiologic hypertrophy may be instrumental in the development of strategies to prevent or reduce the detrimental impact of pathological hypertrophy.

MicroRNAs (miRs or miRNAs) are a class of small non-coding RNAs that regulate gene expression by inducing mRNA cleavage or by inhibiting protein translation [6]. Similar to protein-coding genes, miR expression is variable: some miRs are constitutively expressed, whereas others are expressed in a cell- or tissue-specific manner [7]. Changes in the expression of miRs have been described in almost all cardiovascular disorders [8], [9], [10], and the specific role of miRs in the genesis of cardiac hypertrophy has received great attention in the last decade. Some studies have revealed pro- or anti-hypertrophic abilities of a set of miRs in cardiomyocyte cultures [11] and in a pressure-overloaded mouse model [12], [13]. Moreover, Fernandes *et al.* [14] suggested that miRs might be involved in experimental cardiac hypertrophy induced by swimming training [14]. The present study was conducted to determine the profile of miR expression in an experimental model of exercise-induced left ventricular hypertrophy, based on a set of miRs already known to be expressed in pathological hypertrophy and on previous microarray analyses. As a result we found a set of miRs with down-regulated and up-regulated expression during the development of physiologic cardiac hypertrophy as demonstrated by variations in the levels of miR-26b, -27a, -143 and -150.

## Methods

### Animals

All animals were treated in accordance with the Guidelines for the Care and Use of Laboratory Animals prepared by the National Academy of Sciences and published by the National Institutes of Health (NIH publication no. 85-23, Revised 1996). The protocol was approved by the Committee on Ethics in Research of the Hospital de Clínicas de Porto Alegre (Project number 09–027). Eight to ten week-old male Balb/c mice were studied and kept at the experimental animal facility in the Research Center of Hospital de Clínicas de Porto Alegre, under light and dark cycles of 12 hours, room temperature ranging from 20–25°C, and water and chow *ad libitum.* The animals were divided into two groups of 10–12 animals: sedentary (SED) and exercise (EXE) groups. Analyses were performed after 7 and 35 days of training.

### Model of Physiological Hypertrophy

Physiological hypertrophy was induced by a standard protocol of voluntary exercise for 35 days, as previously described [15], [16]. In brief, animals were kept in cages with metal wheels (with diameters of 12 cm) where they could perform voluntary exercise. Each cage contained four mice and a wheel for each animal. Odometers were installed in each cage to obtain the following data related to exercise load: daily distance (m), average speed (m/min), maximum speed (m/min) and running time (hours). These measures were reviewed and recorded by an investigator every 24 hours during the protocol. The animals in the sedentary group were kept in standard cages without exercise wheels. Subsets of animals were sacrificed at 7 and 35 days after the initiation of the protocol.

### Echocardiography

Animals underwent transthoracic echocardiography at a baseline evaluation and at 7 and 35 days after training, without the use of anesthesia. The echocardiograms were performed by an operator trained in human and experimental echocardiography with commercially available equipment (EnVisor HD System, Philips Medical, Andover, MA, USA), with a 12–13 MHz linear transducer and at 2 cm depth imaging. At least three high-quality M-mode tracings of the short-axis view of the left ventricle were captured and stored for off-line analysis. The echocardiographic operator was blinded to the group allocation at all times. The left ventricular diastolic and systolic transverse dimensions were subsequently measured for at least three beats per animal to estimate the left ventricular mass. The left ventricular mass was calculated using the following formula: [1,055*(LVSTd+LVdD+PWTd)^3^–LVDd^3^] [17], where LVSTd represents the left ventricular septal thickness during the diastole, LVdD represents the left ventricular diastolic diameter, and PWTd represents the posterior wall thickness during the diastole.

### Maximal Exercise Test

Mice were submitted to exercise testing on a motor treadmill (Space Saver Treadmill®, USA) at baseline and 7 and 35 days after training to evaluate the individual maximal exercise capacity and to calculate the expected improvement in functional capacity in the EXE group. All animals underwent a 5 minute adaptation period on the treadmill at a speed of 7.7 m/min before the test. The test started with the treadmill at a speed of 15 m/min and the speed was increased by 3 m/min every 2 min at 0% grade of inclination until 45 m/min or exhaustion. The total distance run by each animal was calculated at the end of the test.

### Heart Weighing and Tissue Collection

After the final echocardiographic assessment, the animals were weighed and anesthetized with xylazine (100 mg/kg) and ketamine (10 mg/kg), followed by surgical chest opening and rapid excision of the hearts. Then, the atriums and the right ventricle were excised to isolate the left ventricle. The left and right ventricles were weighed to calculate the left ventricle to body weight ratio (LV/BW in mg/g). After weighing, a tissue sample from the left ventricle of each animal was placed into RNA later® (Qiagen, USA) solution or immediately frozen in liquid nitrogen. Total RNA and miRs were extracted with a miRNAeasy mini kit (Qiagen, USA), according to the manufacturer’s instructions. After extraction, 50 pM of synthetic microRNA-39 from *Caenorhabditis elegans* (cel-miR-39, Qiagen, USA) was added to each sample as a standard control for quantitative real time PCR (qRT-PCR), as previously recommended and validated [18], [19]. Total RNAs and the portion that was enriched for miRs were stored at −80°C for subsequent molecular analyses. The concentration of RNA was determined by a NanoDrop ND-1000 Spectrophotometer (NanoDrop Tech., DE).

### MiR Microarray

A fraction of the total RNA was sent to LC Sciences (LC Sciences, Houston, TX) to perform 4 miR microarrays. We performed this analysis on the miRs collected from a pool of four animals from each group (SED 7 days, EXE 7 days, SED 35 days and EXE 35 days). To generate the RNA pools for each group, RNA was used from 2 mice with the lowest and 2 with the highest LV/BW ratios. The total RNA concentration was normalized among animals. The RNA pools were suspended in a 300 µL of precipitation solution (3 M NaOAc, pH 5.2 and 100% ethanol) and stored at −80°C until shipment. The miR microarray was performed using the miRBase version 16, which allowed for the screening of 1,040 mature mouse miRs.

### Quantitative Real Time PCR (qRT-PCR)

qRT-PCRwas conducted for miRs that had been previously described to be involved in pathological cardiac hypertrophy(miR-21 and miR-195) and in models of cardiovascular disease associated with cardiomyocyte injury (miR-499). Additionally, selected miRs that were significantly altered in our microarray, such as miR-26b, miR-27a, miR-143, miR-150, miR-328, miR-341*, miR-680 and miR-1224, were validated.

The reverse transcription (RT) reactions were run in a Veriti™ 96-Well Thermal Cycler according to the manufacturer’s instructions with a miR Reverse Transcription Kit® (Applied Biosystems Inc., USA). Modified miRs were validated using the TaqMan® miR Expression Assays probes (Applied Biosystems Inc., USA).

MiRqRT-PCR reactions were run in triplicate with a 7500 Real-Time PCR System (Applied Biosystems, Inc., USA). The relative expression of each miR was calculated with the comparative threshold cycle (2^−ΔΔCt^) method [20].

### Prediction of miRNA Targets

We used the TargetScan database (release 6.2 - November 2013) [21] to determine the predicted targets of miR-26b, -27a, -143 and -150 by searching for the presence of conserved 8 mer and 7 mer sites that match each miRNA. Table S1 provides the human orthologs of all predicted target genes: 747 conserved targets for miR-26b (832 conserved and 248 poorly conserved sites); 1003 conserved targets for miR-27a (1098 conserved and 440 poorly conserved sites); 276 conserved targets for miR-143 (289 conserved and 105 poorly conserved sites) and 201 conserved targets for miR -150 (207 conserved and 109 poorly conserved sites).

### Functional Annotation Analysis

Functional annotation analysis was conducted using DAVID tools [22] to query KEGG pathways enriched with predicted miRNA targets. The analyses were conducted using the “fuzzy clustering algorithm” in order to reduce the redundancy among functionally related pathways that share similar target genes. Terms with Benjamini-corrected enrichment p-values <0.01 and FDR <0.05 are provided in Table S2. The association map summarizing the enriched pathways is generated in R/Bioconductor [23] using the software package RedeR [24]. The association map provides a graph representation that reflects the relationships between the terms based on the similarity of their target genes.

### Preliminary Target Gene Analysis

Based on bioinformatic data, we performed preliminary analysis to evaluate potential target genes that could be related to LVH, as insulin-like growth factor 1 receptor (IGR1R), nuclear factor of activated T-cells, cytoplasmic, calcineurin-dependent 1 (NFAT1C), GATA Binding Protein 4 (GATA4), cellular homolog of Myb Avian Myeloblastosis Viral Oncogene Homolog (c-Myb), and Glycogen Synthase Kinase 3 Beta (GSK3B). For target gene analysis, first-strand cDNA samples were synthesized from total RNA using a High Capacity cDNA Reverse Transcription kit (Applied Biosystems), according to the manufacturer’s instructions. RT-qPCR were performed in StepOne™ Real-time PCR System, using Taqman gene expression assays (both from Applied Biosystems Inc, USA), following the manufacturer’s instructions. Gene expression was normalized for glyceraldehyde 3-phosphate dehydrogenase gene (GAPDH). The primers of these genes were tested for their efficiency in the qRT-PCR reaction, which was close to 100%. Target genesqRT-PCR reactions were also run in triplicate with a 7500 Real-Time PCR System (Applied Biosystems, Inc., USA). The relative expression of each gene was calculated with the comparative threshold cycle (2^−ΔΔCt^) method [20].

### Statistical Analysis

All values are expressed as the mean ± SD or SEM. A Student’s t-test or Mann-Whitney test was used for two-group comparisons. Comparisons of parameters among three or more groups were analyzed by a one-way ANOVA for single factors, followed by Bonferroni’s correction for multiple comparisons. A two-tailed p value <0.05 was considered statistically significant. Based on microarray data, we selected to validate miRs that had at least a 3-fold difference in expression (either up-regulation or down-regulation) between the exercise group and the sedentary group. These differences in expression were considered relevant if the p value <0.01 and for miRs that had a fluorescent signal >500 in the microarray. Statistical analyses were performed using SPSS version 18 for Windows.

## Results

### Exercise Load and the Maximal Exercise Test

The animals in this study underwent a 5 week protocol of voluntary wheel running. Table 1 describes the weekly and mean data collected about the exercise load. Overall, the animals ran approximately 5 hours per day during the entire duration of the protocol. The mean daily distance the animals ran increased weekly and peaked at the third week (7.6±3.4 km/day; p = 0.005). Similarly, the mean speed increased over time, with the highest speed achieved at the third and fourth weeks (p<0.001). There was no difference in the maximal speed over the experimental period.

**Table 1:** Exercise measurements for the 5 weeks of voluntary exercise.

Week	Time (h)	Distance (km)	Mean Speed (m/min)	Maximal Speed (m/min)
**1**	05∶06±03∶03	5.1±3.2	15.8±2.3	52.4±14.5
**2**	05∶11±02∶03	6.1±3.2	19.2±3.1	53.7±16.2
**3**	05∶43±02∶15	7.6±3.4	21.8±3.4	60.6±17.3
**4**	05∶13±01∶35	6.9±2.7	21.6±3.1	54.4±12.2
**5**	05∶00±02∶12	6.2±2.8	20.5±2.5	54.5±11.3
**6**	*05∶13±02∶25*	*6.3±3.1*	*19.4±3.7*	*54.9±14.6*
**P value**	0.77	0.005	<0.001	0.14

**Figure 1:**
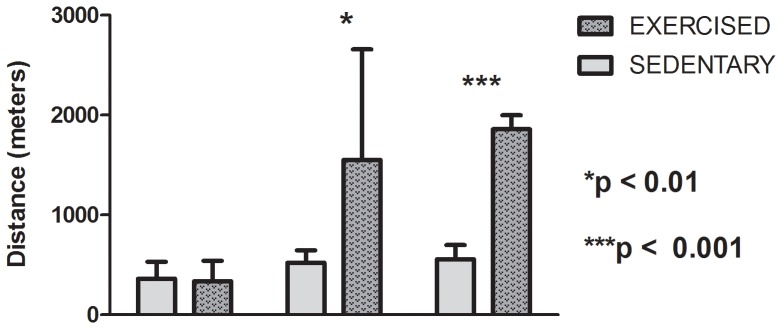
The performances of exercised and sedentary mice in the maximal exercise test. P values represent post-hoc analysis comparing groups between days 7 and 35.

The performance of mice in the maximal exercise testing on the treadmill at baseline and after 7 and 35 days of training is depicted in Figure 1. The animals in the EXE group showed the expected improvement in functional performance compared to the control group, as evaluated by the total running distance before exhaustion. This difference was apparent at day 7 (1550±108 m versus 522±124 m, respectively) and increased further after 35 days of training (1858±141 m versus 557±141 m, respectively).

### Left Ventricular Hypertrophy

Table 2 summarizes the findings related to LVH development during the running protocol. The LV/BW ratio was 7% greater on day 7 and 11% greater on day 35 of training in the EXE group compared with the SED group. The echocardiography-based data demonstrated a similar pattern: the LVSTd was increased by 24% and 32%, whereas the PWTd was increased by 19% and 17% at days 7 and 35, respectively. The estimated left ventricular mass was significantly increased at day 35 in the EXE animals compared with the SED animals.

**Table 2:** Left ventricular weight/body weight (LVW/BW) ratios and echocardiographic data for exercised and sedentary mice at 7 and 35 days of the study.

	7 DAYS		35 DAYS	
	SED	EXE	SED	EXE
**LVW/BW (mg/g)**	4.47±0.16	4.84±0.17 *	4.54±0.13	4.98±0.31*
**PWTd (mm)**	0.74±0.02	0.87±0.08 *	0.75±0.08	0.88±0.04*
**LVSTd (mm)**	0.67±0.05	0.83±0.05 *	0.65±0.07	0.86±0.05*
**LVdD (mm)**	2.51±0.30	2.25±0.29*	2.44±0.40	2.14±0.36*
**Left Ventricular mass (mg)**	44.8±5.8	50.0±6.8	33.5±15.9	58.2±5.0*

Mean and SD for each time point are shown. A Student’s t-test was used to compare the groups.

*indicates p<0.05 when compared with the sedentary group.

### MiR Microarray

**Figure 2:**
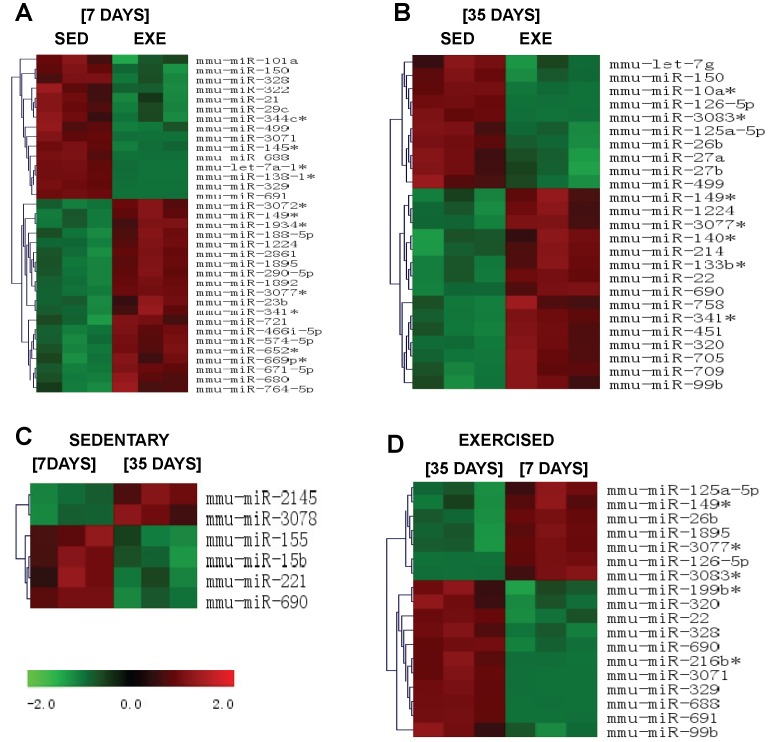
Microarray analysis of miRs in exercised and sedentary mice at days 7 and 35. The green boxes represent down-regulated miRs, and the red boxes represent up-regulated miRs, compared with controls. (A): Comparison of sedentary (left 3 columns) and exercised (right 3 columns) animals at day 7. (B): Comparison of sedentary (left 3 columns) and exercised (right 3 columns) animals at day 35. (C) Comparison of sedentary animals at days 7 (left 3 columns) and 35 (right 3 columns). (D) Comparison of exercised animals at days 7 (left 3 columns) and 35 (right 3 columns). All comparisons have p values <0.01.

The microarray analysis was performed for both groups (EXE and SED) at two time points (days 7 and 35). The data in Figure 2A–D represent 3 technical replicates for each pool analyzed. The day 7 evaluation was conducted to evaluate the early cardiomyocyte adaptations to exercise. At 7 days of training, the microarray identified 35 miRs that were significantly different between the two groups: 20 had an increase in their expression and 15 were down-regulated in EXE group (Figure 2A, p<0.01). At day 35 of training, 25 miRs were different between the groups: 15 were up-regulated and 10 were down-regulated in the EXE group compared with the SED group (Figure 2B, p<0.01). We analyzed the temporal variation in the miR expression profile between the SED groups. We detected only 6 miRs that were differently expressed: 2 miRs were down-regulated and 4 miRs were up-regulated on day 7 compared with day 35 (Figure 2C; p<0.01). Finally, Figure 2D shows the comparison of miR expression in both trained groups at different time points: we detected 7 miRs that were down-regulated and 11 miRs that were up-regulated at 35 days compared with 7 days after training (p<0.01). Detailed information about the microarray analysis can be found in Table S3. These data have been deposited in NCBI’s Gene Expression Omnibus (Martinelli et al., 2013) and are accessible through GEO Series accession number GSE52278 (http://www.ncbi.nlm.nih.gov/geo/query/acc.cgi?acc=GSE52278).

### Microarray Validation by qRT-PCR

**Figure 3:**
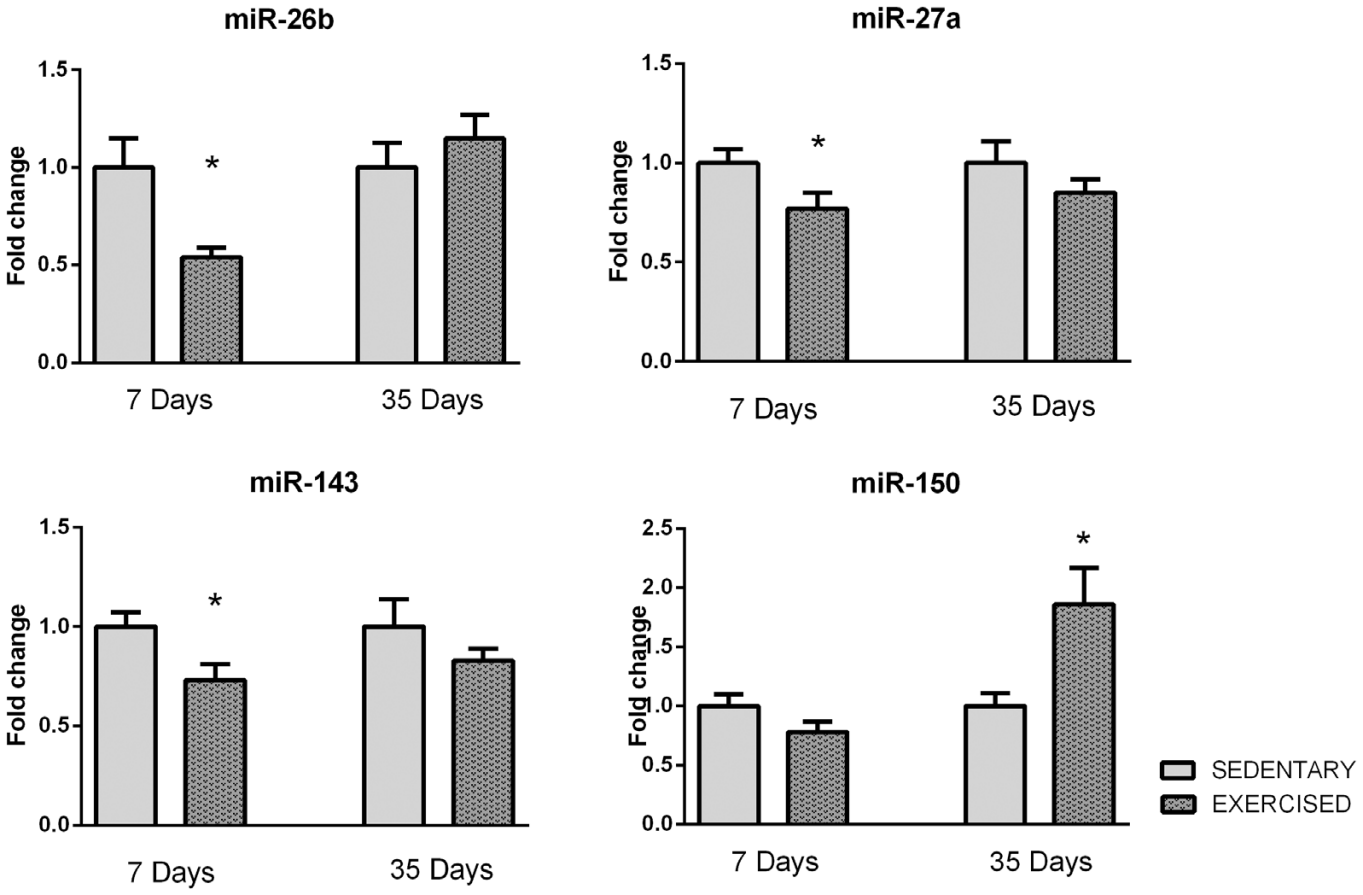
qRT-PCR analysis of miR-26b, -27a, -143 and -150 in exercised and sedentary animals at days 7 and 35 after the start of the study. Data are presented as the mean ± SEM.

All of the qRT-PCR validation data are depicted as relative to SED group at the same time point. As shown in Figure 3, the expression levels of several miRs were significantly decreased, including miR-26b (0.46±0.17, p = 0.02), miR-27a (0.77±0.27, p = 0.03) and miR-143 (0.73±0.28, p = 0.02), in the exercised group at day 7. Furthermore, we detected a remarkable increase in miR-150 expression at 35 days (1.87±0.31, p = 0.01) of training.

**Figure 4:**
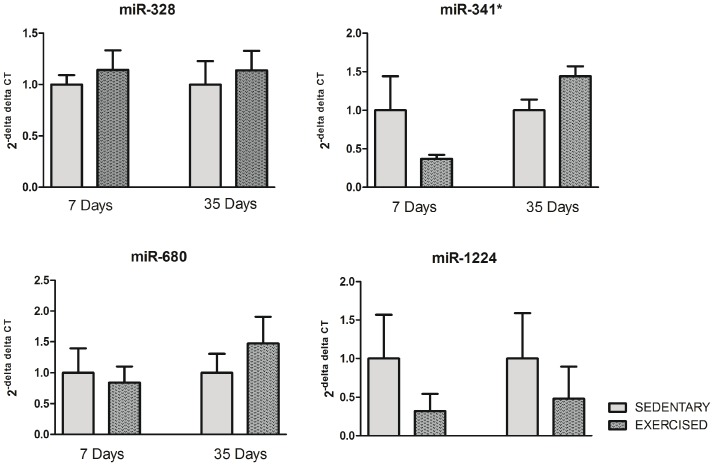
qRT-PCR analysis of miR-328, -341*, -680 and -1224 in exercised and sedentary animals at days 7 and 35 after the start of the study. Data are presented as the mean ± SEM.

**Figure 5:**
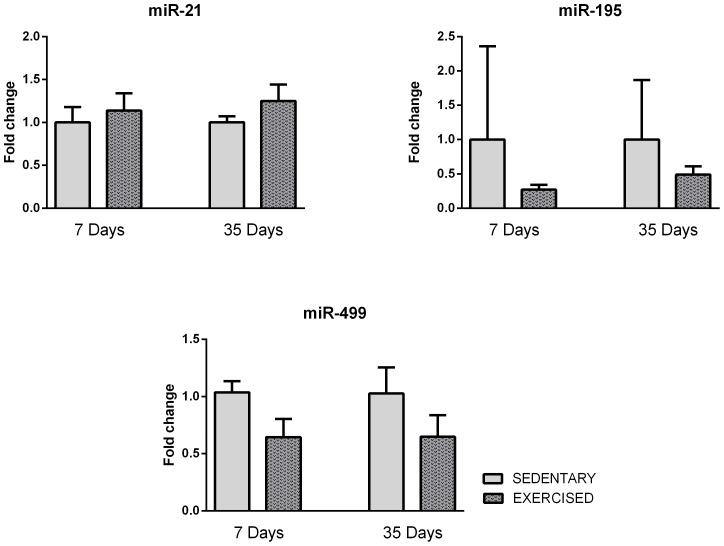
qRT-PCR analysis of miR-21, -195 and -499 in exercised and sedentary animals at 7 and 35 days of exercise.

As shown in Figure 4, we could not confirm the microarray expression data for the expression levels of miR-328, -341*, -680 or -1224. We also analyzed the expression of miRs that have been associated with pathological cardiomyocyte growth, including miR-21, -195 and -499 [25], [26], [27], [28], [29], [30]. We did not observe any differences in the expression levels of miRs -21, -195 and -499 between the EXE and SED groups at any of the time points (Figure 5).

### Signaling Pathways Enriched with the Predicted miRNA Targets

**Figure 6:**
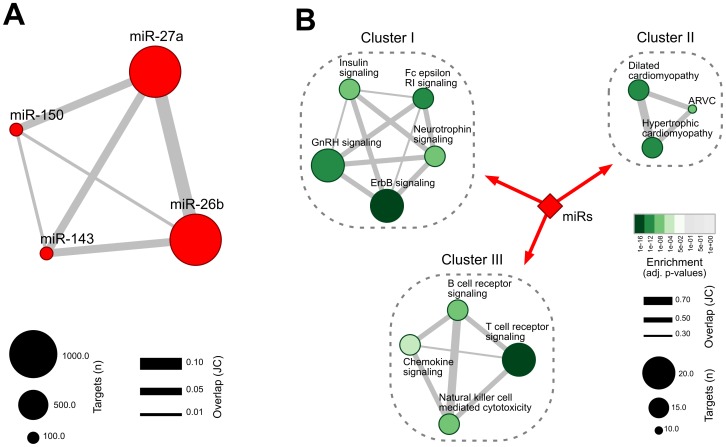
Signaling pathways enriched with predicted miR targets. (A) Graph representation of all targets predicted by the TargetScan for miR-26b, -150, -27a and -143. Node size represents the number of targets inferred for each miR, while edge width corresponds to the overlap between them assessed by the Jaccard coefficient (JC). (B) Association map predicted by the DAVID fuzzy clustering algorithm showing the degree of similarity among KEGG pathways enriched with the miR targets. Different intensities of green indicate the Benjamini-corrected enrichment p-values (FDR <0.05). Node size represents the number of miR targets in each pathway and edge width corresponds to the overlap between functionally related pathways that share similar target genes.

Putative targets of miR-26b, -27a, -143 and -150 were identified by querying the TargetScan database [21] (Table S1). Selecting for target genes related to site conservation resulted in 1938 putative target genes, most of them associated with miR-27a (1003 targets) and miR-26b (832 targets). Figure 6A shows the distribution and the overlap among the inferred targets for each miRNA.

To better understand the predicted interactions, the predicted targets are subjected to functional analyses using DAVID tools [22] to query KEGG pathways enriched with the miR targets. Table S2 lists the enriched KEGG pathways grouped into three functionally related clusters. Figure 6B depicts these clusters in a graph format, representing the distribution of the miR targets annotated for each pathway. Different intensities of green indicate the enrichment p-values, node size represents the number of miR targets in each pathway and edge width corresponds to the overlap between functionally related pathways that share similar target genes. Accordingly, the most enriched pathways are: *ErbB signaling* in *Cluster-I*, *Hypertrophic cardiomyopathy* in *Cluster-II* and *T cell receptor* in *Cluster-III*.

### Preliminary Target Identification

**Figure 7:**
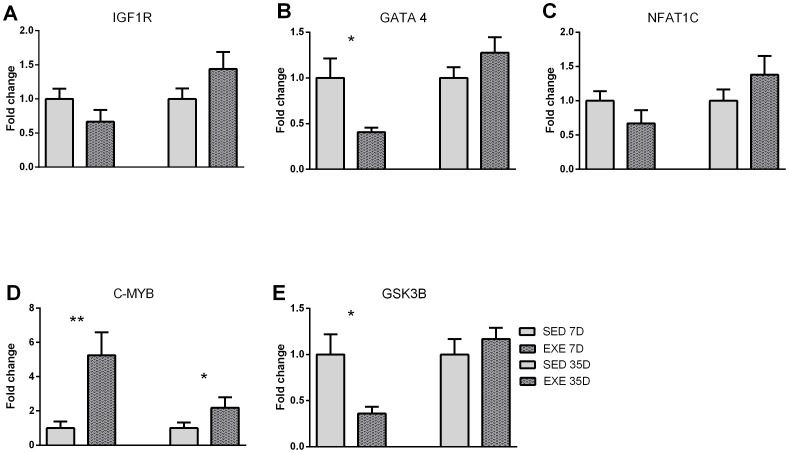
qRT-PCR analysis of potential target genes IGFR1 (A), NFAT1C (B), GATA4 (C), c-Myb (D) and GSK3B (E) in exercised and sedentary animals at days 7 and 35 after the start of the study. Data are presented as the mean ± SEM.

Based on miRBase target prediction, we analyzed the expression levels of microRNAs target genes by qRT-PCR (Figure 7). We did not find significant changes regarding mRNA levels of IGFR1 and NFAT1C gene expression at both time points (Figure 7A and 7C, respectively). We observed a significant reduction of GATA4 levels 7 days after exercise (p = 0.02; Figure 7B) that was not maintained on day 35. As miR-150 was the most up-regulated microRNA, we analyzed two different potential targets: GSK3B and C-Myb. C-Myb was up-regulated both at 7 days (p = 0.002) and 35 days after exercise (p = 0.04; Figure 7D), while GSK3B mRNA levels were decreased only at day 7 (p = 0.01; Figure 7E).

## Discussion

Although a variety of miRs have been identified as mediators of pathologic cardiac hypertrophy, the pattern of miR expression involved in cardiomyocyte growth after a physiological stimulus such as exercise has not been fully elucidated. In the present study, animals that underwent physical training developed the expected physiological LVH, as demonstrated by a significant increase in the left ventricular mass index, which is based on the measured ventricle weight and echo-based parameters. Voluntary exercise also resulted in a considerable improvement in the distance the animals could run in the maximal exercise test. These functional and morphological changes in the left ventricle were paralleled by a distinct pattern of miR expression that did not resemble the profiles previously reported in models of pathological LVH. In particular, we did not find altered levels of miR-195, -499, -341*, -328, -680 and -1224. Importantly, other miRs were significantly altered between the sedentary and exercised groups at different time points.

The role of miR-21 in cardiovascular system has been evaluated by several studies [31], [32], [33], [34]. After an ischemic preconditioning, miR-21 was up-regulated and protected against the ischemic-reperfusion injury by reducing cardiac cell apoptosis via its target gene PDCD4 [8]. Additionally, overexpression of miR-21 decreased H2O2-induced cardiac myocyte death and apoptosis, an effect also mediated by PDCD4 regulation [32]. In the present protocol, we did not observed an increase in miR-21 levels after spontaneous exercise.

We observed a significant reduction in miR-26b expression at 7 days after training began. The target genes of miR-26b predicted by TargetScan (http://www.targetscan) are related to pro-survival pathways, such as the insulin-like growth factor 1 (IGF-1) and PI3K regulatory subunit gamma pathways, both of which are key signals in adaptative hypertrophy [35]. Although Jentzsch *et al.* [11] did not identify miR-26b as a pro-hypertrophic miR, our data are in accordance with a recent report demonstrating that the down-regulation of miR-26b in the heart is required for cardiac hypertrophy induced by pressure overload [36].

Our study identified a remarkable increase in miR-150 expression in the exercised groups after 35 of training. Previous studies evaluating the role of miR-150 in pathologic LVH have found diverse functions. Presumed cardiomyocyte hypertrophy induced by experimental diabetes was recently found to be associated with the reduced expression of miR-150 and increased expression of p300, a transcriptional co-activator with histone acetyl transferase activity [31]. Thoracic aortic banding was reported to down-regulate mir-150 in three studies [26], [28], [37]. Moreover, forced overexpression of miRs that were down-regulated in cardiomyocytes caused an apparent reduction in cell size, suggesting that some miRs normally function to suppress growth and are therefore down-regulated to enhance hypertrophy [28]. We must bear in mind, however, that the initial stimuli, whether physiological or pathological, may be a key factor in determining the pathways in which a specific miR will function [35]. For instance, recent studies have reported potential regulatory roles for miR-150 in growth and differentiation in various cell lineages. The increased expression of miR-150 in cancer epithelial cells decreases P2X7 mRNA levels through the activation of the miR-150 instability target sites located at the 3′-UTR-P2X7 [38]. The P2X7 receptor regulates a pro-apoptotic pathway that modulates cell growth and is post-transcriptionally down-regulated in cancer epithelial cells. The up-regulation of miR-150 during cell differentiation and proliferation implies that it fulfills a functional role in cell division or, in the case of cardiomyocytes, cell growth. Previous reports also found an anti-growth function for miR-150 inhibitors in cervical and lung cancer-derived cell lines [39]. Wu *et al*. observed an increased expression of miR-150 levels in gastric cancer tissue lines, and forced over-expression of miR-150 promoted the proliferation of gastric cancer cells, whereas suppression of miR-150 with antagomirs had the opposite effect. Interestingly, miR-150 was found to directly target the pro-apoptotic gene EGR2 at the translational level [40]. Overall, taken together with the evident up-regulation of mir-150 observed in our protocol of physiological LVH, these findings agree with the hypothesis that the regulation of miRs is a dynamic process that depends on the cellular microenvironment and the observed cellular changes most likely reflect the combined actions of multiple miRs [26], [40].

The miR-27 family has been implicated in the development of cardiac hypertrophy, although it remains unclear how these molecules modulate growth of cardiomyocytes in response to different stimuli. We detected a reduction in miR-27a levels at 7 days of training; this finding is in agreement with recent data from Jentzsch *et al*. [11]. These authors tested a comprehensive library of synthetic miRs to identify which miRs are pro- or anti-hypertrophic factors using a novel microscopy-based automated assay with an edge detection algorithm to assess cardiomyocyte size. Only 3 miRs were found to have anti-hypertrophic potential (miR-27a, -27b and -133) [11]. MiR-27a has been implicated in carcinogenesis, angiogenesis and endothelial cell repulsion by targeting semaphorin 6A [41]. In addition, miR-27 has been implicated in the regulation of several tumor suppressors, such as FBW7, which is involved in cyclin E degradation and cell cycle progression [42], and FOXO1, which is a transcription factor that controls the genes involved in the apoptotic response and cell cycle checkpoints in breast cancer cells [43]. Interestingly, Fernandes *et al*. [14] have shown an increase in miR-27a and -27b expression in the hearts from rats subjected to a forced swimming protocol. We detected a reduction in miR-143 expression at 7 days after training initiation in the exercised group, and these data are in agreement with previously published results [14]. Duration, intensity and willingness to perform exercise (spontaneous wheel running versus forced swimming) are intrinsic differences between our training protocols and others. Our protocol involved less intense but longer duration exercise, which is a stimulus that can have different impacts on intracellular pathways that regulate cell growth and apoptosis.

Our approach to identify potential miR targets revealed relevant pathways related to cell survival and hypertrophy development. We uncovered 3 major clusters containing more than a single pathway, as demonstrated in Figure 6. The ErbB (cluster I) and T cell receptor signaling (cluster III) pathways were the ones with the most number of targets and overlaps. Sysa-Shah et al. [44] demonstrated that cardiac-restricted over-expression of ErbB2 in transgenic mice led to the development of striking concentric cardiac hypertrophy. Increased ErbB2 over-expression in the heart also activated protective signaling pathways, involving phosphoinositide 3-kinase (PI3K)/AKT and leading to an anti-apoptotic shift in the heart. Previous studies have suggested that the PI3K/AKT pathway is directly involved in the induction of physiological, but not pathological, cardiac hypertrophy [44], [45]. Insulin-related pathways (cluster I) are also associated with the susceptibility to cause physiologic cardiac hypertrophy by over-expression of insulin-like growth factor 1 (IGF-1) and insulin-like growth factor 1 receptor (IGF1R), via the PI3K (p110alpha) pathway [46], [47]. PI3K p110 alpha is a key mediator of T cell receptor signaling, regulating both T cell activation and migration of primed T cells to non-lymphoid antigen-rich tissue. Ying et al. [48] have recently shown that suppression of p110alpha activity significantly attenuates the development of chronic rejection of heart grafts, by impairing the localization of antigen-specific T cells to the grafts.

We have performed preliminary analysis of five potential gene targets based on the miRBase target prediction software (IGFR1, GATA4, NFAT1C,c-Myband GSK3B). Sufficiency of GATA4 has been traditionally linked to a hypertrophic phenotype in vitro and in vivo [49]. Surprisingly, our data suggest a down-regulation of GATA4 (a predicted target of miR-27a) early after initiation of spontaneous exercise, an intriguing finding that deserves further investigation, but suggests that pathological and physiological LVH might involve different intracellular pathways. We also demonstrated a significant decrease in GSK3-beta (a predicted target of miR-150) in the early period of training.GSK-3 kinases have been reported to negatively regulate several transcription factors and basic cell cycle regulators implicated in heart development. Kerkela et al have demonstrated that GSK3B knockout mice (−/−) embryos develop a hypertrophic myopathy caused by cardiomyocyte hyperproliferation that was associated with increased expression and nuclear localization of three regulators of proliferation (GATA4, cyclin D1, and c-Myc) [50]. The unexpected up-regulation of C-Myb (a proto-onco gene with proliferative effects anda recognized target of miR-150) [51], at both time points (day 7 and day 35), may represent a regulation effect based on the interaction of other miRs (*off target*).

Some limitations related to our study design must be considered. The microarray analysis was performed with miRs isolated from tissues from a pool of four animals in each group and time period with the assumption that the intrinsic variability in miR expression would be low among animals from the same group. This assumption was subsequently proven not to be completely true, even in animals that developed clear exercised-induced ventricular hypertrophy. In that sense, this variability may be the reason why genes found to be differentially expressed on microarray data were not validated by real time PCR. On the other hand, it is possible that existing differences have been overlooked due to these same reasons.

## Conclusions

Our results elucidate the importance of studying the expression levels of various miRs during the development of physiologic cardiac hypertrophy, as demonstrated by variations in the levels of miR-26b, -27a, -143 and -150. Moreover, our data on miR expression after 7 and 35 days of voluntary exercise in mice suggest that the previously established regulatory pathways controlling pathological hypertrophy are not deregulated in physiologic cardiac growth. Thus, further studies are warranted to validate the targets of these miRs and to determine their functions, which will eventually allow for an understanding of the role of these miRs in cardiomyocyte growing and heart adaptation.

## Supporting Information

Table S1 **Human orthologs of all predicted target genes.** 747 conserved targets for miR-26b (832 conserved and 248 poorly conserved sites); 1003 conserved targets for miR-27a (1098 conserved and 440 poorly conserved sites); 276 conserved targets for miR-143 (289 conserved and 105 poorly conserved sites) and 201 conserved targets for miR -150 (207 conserved and 109 poorly conserved sites).
(XLSX)

Table S2 **Predicted targets subjected to functional analyses using DAVID tools to query KEGG pathways enriched with the miR targets.**
(XLSX)

Table S3 **Detailed information about the microarray analysis.** These data have been deposited in NCBI’s Gene Expression Omnibus (Martinelli et al., 2013) and are accessible through GEO Series accession number GSE52278.
(DOCX)
