# Supplementary material for: An Analysis of the Global Expression of MicroRNAs in an Experimental Model of Physiological Left Ventricular Hypertrophy
Source: PLoS One. 2014 Apr 21;9(4):e93271. doi: 10.1371/journal.pone.0093271 (PMC3994002; doi:10.1371/journal.pone.0093271)
Supplement: Table S3 — Detailed information about the microarray analysis. These data have been deposited in NCBI’s Gene Expression Omnibus (Martinelli et al., 2013) and are accessible through GEO Series accession number GSE52278. (DOCX) [file pone.0093271.s003.docx]

Table 1. Microarray expression data analysis at 7 days after exercise. Statistical analysis performed with T-test.

|  |  |  | Group 1 | Group 2 |  |
| --- | --- | --- | --- | --- | --- |
|  |  |  | **Control 7 days** | **Exercise 7 days** | **Log2 (G2/G1)** |
| No. | Reporter Name | p-value | Mean | Mean |  |
| 757 | mmu-miR-466i-5p | 4.39E-04 | 960 | 2,781 | 1.53 |
| 907 | mmu-miR-671-5p | 7.10E-04 | 623 | 2,192 | 1.81 |
| **63** | **mmu-miR-1224** | **7.69E-04** | **451** | **2,070** | **2.20** |
| 849 | mmu-miR-574-5p | 8.64E-04 | 395 | 863 | 1.13 |
| 145 | mmu-miR-149* | 1.03E-03 | 15,709 | 28,544 | 0.86 |
| 478 | mmu-miR-3072* | 1.08E-03 | 508 | 1,353 | 1.41 |
| 401 | mmu-miR-290-5p | 1.22E-03 | 131 | 552 | 2.07 |
| 198 | mmu-miR-1895 | 1.48E-03 | 662 | 1,555 | 1.23 |
| 489 | mmu-miR-3077* | 1.54E-03 | 1,807 | 8,137 | 2.17 |
| 862 | mmu-miR-652* | 1.64E-03 | 988 | 2,660 | 1.43 |
| 238 | mmu-miR-1934* | 2.10E-03 | 860 | 3,015 | 1.81 |
| **146** | **mmu-miR-150** | **2.23E-03** | **2,042** | **1,086** | **-0.91** |
| 193 | mmu-miR-188-5p | 2.93E-03 | 366 | 1,019 | 1.47 |
| 430 | mmu-miR-29c | 2.95E-03 | 2,850 | 1,108 | -1.36 |
| 194 | mmu-miR-1892 | 3.05E-03 | 1,035 | 5,292 | 2.35 |
| **812** | **mmu-miR-499** | **3.12E-03** | **10,246** | **6,389** | **-0.68** |
| **923** | **mmu-miR-680** | **3.40E-03** | **174** | **572** | **1.71** |
| **611** | **mmu-miR-341*** | **4.52E-03** | **952** | **2,205** | **1.21** |
| 381 | mmu-miR-23b | 5.04E-03 | 14,340 | 18,718 | 0.38 |
| 579 | mmu-miR-322 | 5.28E-03 | 746 | 434 | -0.78 |
| **339** | **mmu-miR-21** | **7.33E-03** | **2,346** | **1,399** | **-0.75** |
| 399 | mmu-miR-2861 | 7.35E-03 | 15,041 | 30,259 | 1.01 |
| Following transcripts are statistically significant but have low signals (signal < 500) | | | | | |
| 932 | mmu-miR-691 | 1.55E-04 | 103 | 0 | -13.65 |
| 133 | mmu-miR-145* | 2.44E-04 | 221 | 41 | -2.45 |
| 118 | mmu-miR-138-1* | 3.91E-04 | 89 | 0 | -13.37 |
| 592 | mmu-miR-329 | 5.40E-04 | 68 | 0 | -13.08 |
| 2 | mmu-let-7a-1* | 8.42E-04 | 101 | 0 | -13.49 |
| 475 | mmu-miR-3071 | 2.29E-03 | 56 | 0 | -12.81 |
| 590 | mmu-miR-328 | 2.37E-03 | 381 | 237 | -0.69 |
| 930 | mmu-miR-688 | 2.93E-03 | 31 | 0 | -11.99 |
| 965 | mmu-miR-721 | 5.41E-03 | 87 | 223 | 1.36 |
| 903 | mmu-miR-669p* | 5.46E-03 | 158 | 324 | 1.03 |
| 620 | mmu-miR-344c* | 6.00E-03 | 81 | 53 | -0.61 |
| 22 | mmu-miR-101a | 9.61E-03 | 175 | 57 | -1.63 |
| 985 | mmu-miR-764-5p | 9.71E-03 | 37 | 172 | 2.22 |

**Table 2. Microarray expression data analysis at 7 days after exercise. Statistical analysis performed with T-test.**

|  |  |  | Group 1 | Group 2 |  |
| --- | --- | --- | --- | --- | --- |
|  |  |  | Control 35 days | Exercise 35 days | Log2 (G2/G1) |
| No. | Reporter Name | p-value | Mean | Mean |  |
| 611 | mmu-miR-341* | 4.77E-04 | 700 | 3,464 | 2.31 |
| 931 | mmu-miR-690 | 8.96E-04 | 8,097 | 10,997 | 0.44 |
| **391** | **mmu-miR-26b** | **1.01E-03** | **6,176** | **1,489** | **-2.05** |
| **146** | **mmu-miR-150** | **1.35E-03** | **2,276** | **967** | **-1.24** |
| 489 | mmu-miR-3077* | 1.53E-03 | 1,354 | 3,576 | 1.40 |
| 729 | mmu-miR-451 | 1.81E-03 | 3,111 | 6,559 | 1.08 |
| 105 | mmu-miR-133b* | 1.91E-03 | 341 | 1,045 | 1.61 |
| 953 | mmu-miR-709 | 1.94E-03 | 17,481 | 28,056 | 0.68 |
| 74 | mmu-miR-125a-5p | 2.21E-03 | 5,081 | 2,243 | -1.18 |
| 63 | mmu-miR-1224 | 3.49E-03 | 559 | 1,269 | 1.18 |
| 350 | mmu-miR-214 | 3.90E-03 | 2,165 | 3,970 | 0.88 |
| 369 | mmu-miR-22 | 4.36E-03 | 2,025 | 3,058 | 0.60 |
| 577 | mmu-miR-320 | 5.12E-03 | 1,915 | 3,701 | 0.95 |
| 1039 | mmu-miR-99b | 5.27E-03 | 957 | 1,676 | 0.81 |
| 145 | mmu-miR-149* | 6.30E-03 | 13,473 | 19,859 | 0.56 |
| **393** | **mmu-miR-27a** | **6.53E-03** | **5,732** | **3,073** | **-0.90** |
| 948 | mmu-miR-705 | 6.66E-03 | 3,542 | 6,375 | 0.85 |
| 812 | mmu-miR-499 | 7.11E-03 | 10,943 | 7,453 | -0.55 |
| 15 | mmu-let-7g | 7.77E-03 | 11,064 | 6,601 | -0.75 |
| 395 | mmu-miR-27b | 7.89E-03 | 7,669 | 4,558 | -0.75 |
| 123 | mmu-miR-140* | 8.91E-03 | 429 | 1,372 | 1.68 |
| Following transcripts are statistically significant but have low signals (signal < 500) | | | | | |
| 37 | mmu-miR-10a* | 1.31E-04 | 59 | 0 | -15.76 |
| 81 | mmu-miR-126-5p | 1.83E-04 | 110 | 0 | -16.09 |
| 501 | mmu-miR-3083* | 1.19E-03 | 36 | 0 | -14.72 |
| 976 | mmu-miR-758 | 6.29E-03 | 21 | 76 | 1.84 |
